# Robust Reductions of Body Weight and Food Intake by an Oxytocin Analog in Rats

**DOI:** 10.3389/fphys.2021.726411

**Published:** 2021-09-27

**Authors:** Clinton T. Elfers, James E. Blevins, Elizabeth A. Lawson, Richard Pittner, David Silva, Alex Kiselyov, Christian L. Roth

**Affiliations:** ^1^Center for Integrative Brain Research, Seattle Children’s Research Institute, Seattle, WA, United States; ^2^VA Puget Sound Health Care System, Office of Research and Development Medical Research Service, Department of Veterans Affairs Medical Center, Seattle, WA, United States; ^3^Division of Metabolism, Endocrinology and Nutrition, Department of Medicine, University of Washington School of Medicine, Seattle, WA, United States; ^4^Neuroendocrine Unit, Massachusetts General Hospital and Department of Medicine, Harvard Medical School, Boston, MA, United States; ^5^OXT Therapeutics, Saint Louis, MO, United States; ^6^Division of Endocrinology, Department of Pediatrics, University of Washington, Seattle, WA, United States

**Keywords:** oxytocin, oxytocin analog, *in vivo* stability, diet-induced obesity, rats, food intake, body weight

## Abstract

**Background:** Oxytocin is a hypothalamic neuropeptide that participates in the network of appetite regulation. Recently the oxytocin signaling pathway has emerged as an attractive target for treating obesity. However, the short half-life limits its development as a clinical therapeutic. Here we provide results from testing a long-lasting, potent and selective oxytocin analog ASK1476 on its efficacy to reduce food intake and body weight in comparison to the native oxytocin peptide.

**Methods:** ASK1476 features two specific amino acid substitutions in positions 7 and 8 combined with a short polyethylene glycol spacer. Short time dose escalation experiments testing increasing doses of 3 days each were performed in diet-induced overweight (DIO) male rats assessing effects on body weight as well as changes in food intake. Furthermore, DIO rats were tested for changes in body weight, food intake, temperature, and locomotor activity over 28 days of treatment (oxytocin, ASK1476, or vehicle).

**Results:** In dose escalation experiments, significant reductions in food intake relative to baseline were detected beginning with doses of 15 nmol/kg ASK1476 (−15.2 ± 2.3 kcal/d, *p* = 0.0017) and 20 nmol/kg oxytocin (−11.2.9 ± 2.4 kcal/d, *p* = 0.0106) with corresponding significant changes in body weight (ASK1476: −5.2 ± 0.8 g, *p* = 0.0016; oxytocin: −2.6 ± 0.7 g, *p* = 0.0326). In long-term experiments, there was no difference on body weight change between 120 nmol/kg/d ASK1476 (−71.4 ± 34.2 g, *p* = 0.039) and 600 nmol/kg/d oxytocin (−91.8 ± 32.2 g, *p* = 0.035) relative to vehicle (706.9 ± 28.3 g), indicating a stronger dose response for ASK1476. Likewise, both ASK1476 and oxytocin at these doses resulted in similar reductions in 28-day cumulative food intake (ASK1476: −562.7 ± 115.0 kcal, *p* = 0.0001; oxytocin: −557.1 ± 101.3 kcal, *p* = 0.0001) relative to vehicle treatment (2716 ± 75.4 kcal), while no effects were detected on locomotor activity or body temperature.

**Conclusion:** This study provides proof-of-concept data demonstrating an oxytocin analog with extended *in vivo* stability and improved potency to reduce food intake and body weight in DIO animals which could mark a new avenue in anti-obesity drug interventions.

## Introduction

Nearly 73 million people in the United States are overweight or obese (Ogden et al., 2012), which is the second leading cause of preventable death, at a cost to the U.S. health-care system of 147 billion dollars per year or 9% of all medical expenditures (Finkelstein, 2014), and by 2030 nearly 1 in 2 adults will have obesity in the US (Ward et al., 2019). Even in children and adolescents an estimated 16.6% have obesity across the US (Imoisili et al., 2021), and accelerating rates are reported form Asian countries (Collaboration, 2017). Diet and exercise can reduce body weight by as much as 10% (Wadden et al., 1999; Zibellini et al., 2016) and improve health (Magkos et al., 2016) over the short-term but results in subpar long-term weight loss (Wu et al., 2009; Barte et al., 2010; Dombrowski et al., 2014). Although pharmacotherapy improves weight loss, often the effect is modest and associated with safety and tolerability issues, e.g., behavioral and gastrointestinal side effects, particularly at more efficacious doses (Azebu, 2014; Fujioka, 2015; Coskun et al., 2018; O’Neil et al., 2018; Tillner et al., 2019). The often modest reductions in body weight are due to compensatory adaptations that increase levels of appetite-stimulating hormones (e.g., ghrelin), decrease levels of appetite-suppressing hormones (e.g., peptide YY released from cells in the ileum and colon in response to feeding or leptin from adipose tissue), and reduced energy expenditure (Hill et al., 2012).

Recently, the oxytocin (OXT) signaling pathway has emerged as an attractive target for treating obesity (Blevins and Baskin, 2015). Mutations in OXT or its receptor (OXTR) are directly associated with obesity in animals (Kasahara et al., 2007; Takayanagi et al., 2008; Camerino, 2009; Zhang et al., 2011; Sun et al., 2019) and humans (Wheeler et al., 2013; Qian et al., 2014; Yuan et al., 2016). OXT is a hypothalamic neuropeptide that participates in the network of appetite regulation (Maejima et al., 2014), as well as many other neurologic processes (Shen, 2015). OXT is produced in the paraventricular nucleus (PVN) and supra-optic nucleus (SON) of the hypothalamus, thus OXT deficiency is plausible in individuals with hypothalamic and pituitary tumors (Gebert et al., 2018; Aulinas et al., 2019). As shown in macaques, only small amounts of peripherally administered OXT are able to enter the CSF from the periphery (Freeman et al., 2016) and penetrating the blood brain barrier may not be required to see central effects (Leng and Ludwig, 2016), specifically reductions in food intake induced by peripheral signal reaching brain via vagus nerve (Ho et al., 2014; Iwasaki et al., 2015). There is now growing evidence that peripheral administration of OXT reduces food intake, enhances energy expenditure, and prevents or reduces weight gain in diet-induced obese and genetically obese rodent models, and reduces food intake and body weight in diet-induced obese non-human primates (Maejima et al., 2011; Morton et al., 2012; Blevins et al., 2015) as well as obese humans (Zhang et al., 2013; Hsu et al., 2018). Intranasal (IN) application of OXT modulates hypothalamic activation to visual food cues (van der Klaauw et al., 2017; Plessow et al., 2018), as well as brain areas involved in reward and cognitive control of food intake in humans (Spetter et al., 2018; Kerem et al., 2020; Plessow et al., 2021).

While these results are encouraging, there are several limitations which have hampered further development of OXT as a weight loss drug. First, due to a short half-life (∼5 min) *in vivo* (Ring et al., 2010; Steckler et al., 2012), clinical studies of intranasal OXT for weight loss have used four-times per day dosing (Zhang et al., 2013). Second, the duration of exposure with OXT in most prior studies was short, and long-term efficacy and safety data are missing (Lawson et al., 2020). Finally, the potential for toxic side effects upon chronic administration at high concentration and/or frequent dosing (> 2 times daily) remains to be addressed. Of potential concern is the interaction of OXT with the vasopressin receptor (VR) system (Wiśniewski et al., 2014). Indeed, OXT has only fivefold greater affinity for the OXTR compared with the V_1a_R (Akerlund et al., 1999). The weight loss effects of IN OXT are seen at supraphysiologic doses, which raises concerns for cardiovascular effects (e.g., tachycardia and heart failure), hyponatremia and, upon potential CNS exposure, anxiety and aggression via crossover effects at VRs in at risk patients.

In this paper we provide results from testing a long-lasting, peripheral acting, potent and selective OXT analog on its efficacy to reduce food intake and body weight in comparison to the native OXT peptide. Additionally, given the increased plasma binding of this OXT analog, these results indicate that peripheral OXT signaling is sufficient to induce anorectic effects necessary for weight loss. We believe that these results provide proof-of-concept data for the design and development of novel OXT analogs with extended *in vivo* stability with the goal to produce robust, reproducible and sustainable weight loss agents in the future.

## Materials and Methods

All procedures performed in rats were approved by the Institutional Animal Care and Use Committee at the Seattle Children’s Research Institute and were in accordance with the NIH Guide for the Care and Use of Laboratory Animals.

Sprague Dawley rats (male, ∼4 weeks of age, 51–75 g body weight) were switched from standard chow to a high fat diet (Research Diets, Diet # D12492, 60, 20, 20%kcal from fat, protein, and carbohydrate, respectively, 5.21 kcal/g; stored at −20°C). The rats were maintained on this diet for approximately 15 weeks prior to the start of experiments to develop a diet-induced overweight (DIO) model. Animals were individually housed on a 12 h/12 h light/dark cycle (lights on at 04:00 h) in a temperature (23°C) and humidity (mean 50%, SD 10%) controlled room. All body weight measurements were made just prior to the start of the dark cycle.

**FIGURE 1:**
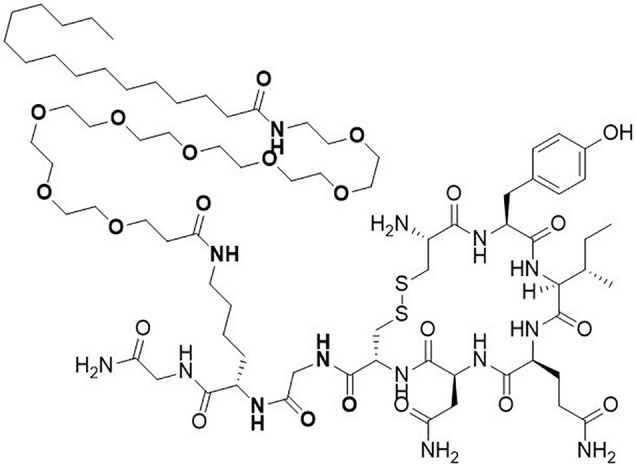
Structure of novel long-acting oxytocin analog ASK1476. The native oxytocin peptide was modified with a substitution of the Leu8 to a Lys appended with a polyethylene glycol space and a palmitoyl group and with a substitution of Gly for the Pro7 to enhance selectivity for the OXT receptor.

The OXT analog ASK1476 (published before as PF-06655075 and PF1) features a specific amino acid substitution changing the Leu^8^ of OXT to a Lys and its subsequent modification with a short polyethylene glycol spacer to a palmitoyl group in order to enhance the plasma proteins binding of the resulting molecule and to decrease the free fraction of the molecule available for metabolism (Figure 1). We further profiled ASK1476 against the vasopressin V2 receptor (V_2_R) (Table 1). V_2_R activation may lead to the risk of hyponatremia along with excessive water intake in the event of excessive or too frequent drug use by patients. The excessive V_1a_R activation may be associated with systemic vasoconstriction or, for local use, at the site of application. To address the OXTR vs. V_1a_R selectivity, Pro^7^ was changed to Gly. Pharmacokinetic (PK) parameters for both OXT (Wiśniewski et al., 2014) and ASK1476 (Modi et al., 2016) were previously described. As evidenced by the PK data, ASK1476 does exhibit the desired increased half-life as well as increased plasma protein binding compared to OXT following both sc and iv administration to rodents suggesting its long-term *in vivo* stability.

**TABLE 1:** Receptor pharmacology data for OXT and ASK1476 (PF1).

Compound	hOXTR, EC_50_ (nM)	hV_1a_R, EC_50_ (nM)	hV_2_R, EC_50_ (nM)	Ratio V2R/OXTR
OXT^a^	2.3	10	7.3	3
ASK1476	0.025^b^	NA (>10,000)^b^	0.36	14

*^a^These data are from Wiśniewski et al. (2014). ^b^These data are from Modi et al. (2016).*

### *In vitro* Functional Assays

V_2_R: Evaluation of the agonist activity of compounds at the human V_2_ receptor expressed in stably transfected CHO cells was conducted by measuring their effects on cAMP production using the HTRF detection method (Cotte et al., 1998).

### Preparation and Administration of Drug

The title compound ASK1476 was synthesized using standard 9-fluorenylmethyloxycarbonyl (Fmoc) solid-phase peptide synthesis methods and purified using HPLC method as described previously (Modi et al., 2016). ASK1476 stock solution was prepared using a 10:1 (wt:wt) Hydroxypropyl-β-cyclodextrin excipient (HP-CD; 1,541 g/mol)/ASK1476 (1,644 g/mol) ratio in sterile ultra-pure H_2_0 at a concentration of 5 mg⋅mL^–1^ and gently agitated at 4°C for 24–28 h; stock solution was stored at 4°C until use. Oxytocin (Syntocinon) was obtained from Selleck Chemical (# P1029; lyophilized) and dissolved in 1 mL of sterile, ultra-pure H_2_O. The stock OXT solution was aliquoted in sterile vials and frozen until day of use. Stock solutions for both drugs were diluted to a working solution in normal saline solution (sterile, injectable) and mixed gently for 30 min at 4°C prior to use. Normal saline solution was used as the vehicle control for all experiments.

Vehicle, native OXT, and ASK1476 treatments were injected once daily at start of dark cycle via subcutaneous injection using a 1 cc 29G insulin syringe for increased dose accuracy. The concentration of both OXT and ASK1476 solutions were adjusted so that dosing volume remained at 1 mL/kg.

### Excipient Effects on Food Intake and Body Weight

As the excipient used to place ASK1476 in solution, HP-CD, was used in excess, a preliminary experiment was run to examine whether it produces any anorectic effects independently. Testing consisted of a 7-day normal saline treated phase followed by a 7-day 2% HP-CD/saline solution (dose 1 mL/kg) treated phase in lean male rats (*n* = 5; at start of HP-CD treatment: mean 410 g, SD 26 g). Given the preparation methods of ASK1476, this dose of HP-CD is comparable to the concentration of free HP-CD that what would occur in a 1 μmol/kg dose of ASK1476 using our drug preparation protocol. Food intake was continuously recorded using AccuScan DietMax (currently OmniTech Electronics, Inc., Diet System) cages and data were binned into 15-min intervals for analysis. The start of the 24-h food intake period aligned to the lights turning off in the animal room. Body weight was recorded, and treatment injections were administered just prior to the start of the dark cycle.

### Dose Escalation

Two groups of male DIO Sprague Dawley rats (*n* = 8 each) were balanced for body weight and food intake profiles in order to establish a dose response relationship for ASK1476 and OXT with respect to those measures. Sequential rounds of a 3-day baseline phase (vehicle treated), a 3-day treatment phase, and a washout phase (2–5 days as necessary) were tested. Doses tested include 6, 15, 30, 60, 120, and 300 nmol/kg (10, 25, 50, 100, 200, and 500 μg/kg) for ASK1476 and 20, 60, 300, 600, and 1200 nmol/kg for OXT. High fat diet was accessible to the rats from all but the last 6 h of the light cycle and water was provided *ad libitum*. This daily period of food restriction was used to prevent incidental feeding in the event animals were disturbed from their sleep by noise from surrounding rooms. To assess a potential pica response (nausea/visceral malaise) in animals, powdered kaolin (Sigma-Aldrich, K1512) was made available on drug treatment days with a restriction period similar to the high fat diet. Prior to the first experiment, rats were provided access to kaolin for 2 days so that they could familiarize themselves with it prior to start of testing. Food and kaolin intake were continuously recorded using AccuScan DietMax (currently OmniTech Electronics, Inc., Diet System) cages. The high fat diet was compressed in the food tray to form a solid block of food. This allows for more accurate meal size measurements by preventing the animals from removing entire pellets at time. All food and kaolin intake data were binned into 15-min intervals for analysis and the start of the 24-h food intake period aligned to the lights turning off in the animal room. Body weight was recorded, and treatment injections were administered just prior to the start of the dark cycle.

### Long-Term Drug Intervention

**FIGURE 2:**
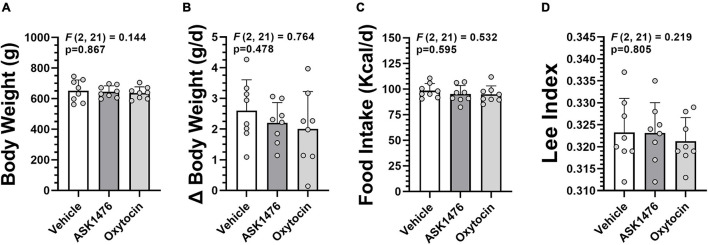
Baseline data used for group assignment in the long-term (28-day) vehicle vs. ASK1476 vs. OXT experiment. Groups were well matched with no difference in body weight at start of treatment **(A)**, average daily body weight change during 7-day baseline phase **(B)**, and average daily food intake during 7-day baseline phase **(C)**. Additionally, Lee Index at start of treatment was used as an indicator of adiposity **(D)**.

Male DIO Sprague Dawley rats (*n* = 24) were generated as outlined above and were 644.5 g (50 g) at the start of drug treatment. The animals were divided into three treatment groups (*n* = 8 each) balanced for body weight, body weight gain during baseline, food intake, and Lee Index [body weight^(1/3)/snout to anus length (mm)] (Lee, 1929; Simson and Gold, 1982) as an indicator of adiposity (Figure 2). In a subset of the animals (*n* = 5 per group, balanced for prior mentioned criteria), Mini-Mitter transponders (PDT-4000 E-Mitter, Respironics Inc.) were implanted posterior to the left scapula at least 9 days prior to start of baseline measures to ensure sufficient recovery time. Body temperature and gross motor activity were continuously recorded via these transponders. Animals were assessed for changes in body weight and food intake over a 7-day baseline phase and 28-day treatment (OXT, ASK1476, or vehicle) phase. High fat diet and water were provided *ad libitum* and intake was recorded with daily hopper and water bottle weighs at time of body weight assessment just prior to drug administration. Blood samples were taken at start of intervention by tail vein bleeding and via cardiac puncture under an isoflurane induced surgical plane of anesthesia at euthanasia. Animals were euthanized on treatment day 29, 4 h after receiving a dose of their assigned treatment, via decapitation under isoflurane anesthesia immediately following blood draw. Body composition was assessed on carcasses.

Treatment doses were selected based on data collected during the dose escalation experiment to achieve an initial estimated 20% reduction in caloric intake relative to baseline (ASK1476 30 nmol/kg, OXT 600 nmol/kg). Doses were then titrated after four (ASK1476 increased to 60 nmol/kg, no change for OXT) and 8 days (ASK1476 increased to 120 nmol/kg, no change for OXT) of treatment to equilibrate the effect of the drugs on caloric intake. From treatment day 8 on the doses were maintained to assess long-term efficacy at a therapeutically meaningful dose.

### Blood Parameters

Commercially available enzyme-linked immunosorbent assays (ELISA) were used for quantitative assessment of leptin (MilliporeSigma, Burlington, MA) and insulin (MilliporeSigma, Burlington, MA); all with intra-assay CV < 4%. Levels of sodium, potassium, glucose, cholesterol (total and HDL), triglycerides, alanine transaminase (ALT), and aspartate transaminase (AST) were determined in serum obtained at sacrifice by cardiac puncture on a Modular P chemistry analyzer (Roche Diagnostics, Germany) at the University of Washington NORC Core, Seattle, WA.

### Body Composition Measurements

Body composition (total and relative fat mass, lean mass, and water content) was assessed on carcasses from the long-term drug intervention study using Quantitative Magnetic Resonance (EchoMRI 4-in-1, Echo Medical Systems, Houston, TX) at the Rodent Metabolic and Behavioral Phenotyping Core at the VA Puget Sound Health Care System (VAPSHCS).

### Statistical Analyses

Statistical analyses were performed using GraphPad Prism Software (la Jolla, CA United States) and STATA (StataCorp LP, College Station, TX). Two-way ANOVA was used for comparison of repeated measures; a mixed effects model was used for repeated measures with missing data. One-way ANOVA was used for cross-sectional comparisons of measures taken at only one time point. A Geisser-Greenhouse correction for non-sphericity was applied as appropriate. *Post hoc* comparisons were made using Dunnett’s Multiple Comparison Test with the vehicle group as a control, Tukey’s Multiple Comparison Test when comparisons were made between all three treatment groups. Simple linear regression analyses were used to assess correlation between two separate measures. Skewed data were log-transformed prior to analysis or a non-parametric test was used, i.e., Kruskal-Wallis. In all instances, a *P* < 0.05 was considered significant. All values are represented as mean (SD).

For comparisons of mean longitudinal changes in body temperature and activity, only the first 12 days of treatment, divided into three 4-day phases, were included in the analysis. These 4-day phases allowed for analyses to consider the ASK1476 dose titration.

## Results

### *In vitro* Functional Assays

Data collected from the cAMP assay indicate that ASK1476 is a potent activator of human V_2_R with an EC_50_ of 0.36 nM (Table 1).

### Excipient Effects on Food Intake and Body Weight

**FIGURE 3:**
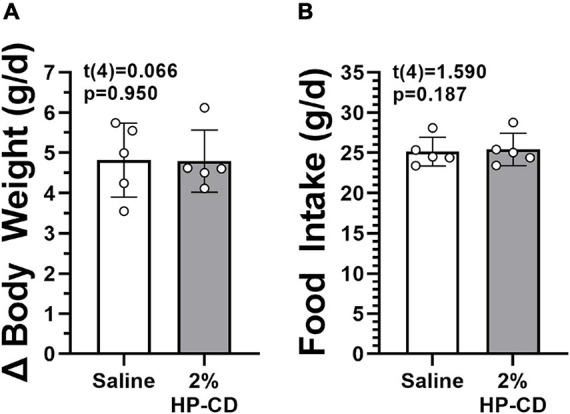
Effect of hydroxypropyl-β-cyclodextrin (HP-CD) on food intake **(A)** and changes in body weight **(B)**. HP-CD is the excipient used to place ASK1476 into solution for injection. No effect of treatment was seen during 7-day saline treatment and 7-day 2% HP-CD/saline solution treatment at 1 mL/kg.

The dose used in this experiment exceeded the unbound HP-CD concentration in every ASK1476 dose tested in the presented experiments. No difference in 7-day food intake (0.26 g, 95% CU −0.19 to 0.71, *p* = 0.187) or body weight gain (−0.02 g, 95% CU −0.99 to 0.95, *p* = 0.950) were detected between saline and 2% HP-CD/saline solution injections (Figure 3).

### Body Weight and Food Intake in Dose Escalation Studies

**FIGURE 4:**
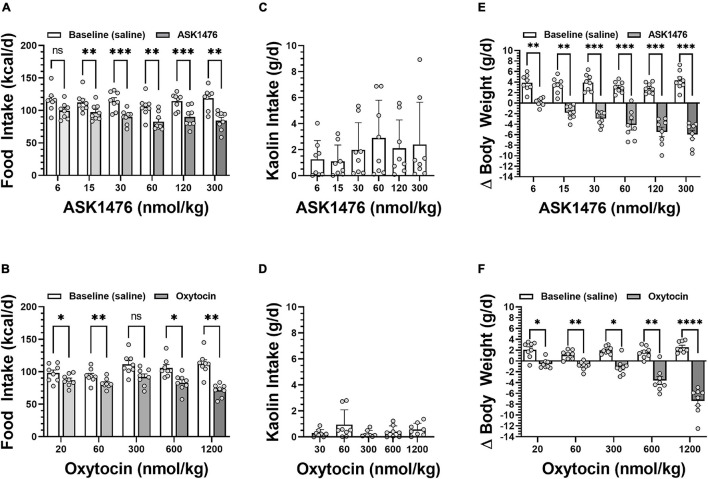
Dose effect of both analog ASK1476 **(A)** and native oxytocin (OXT) **(B)** on food intake, kaolin intake, and body weight in DIO rats. ASK1476 showed a comparable reduction of food intake following injection of 30 to 600 nmol/kg of oxytocin demonstrating stronger potency. While kaolin intake, a marker for nausea and visceral malaise induction in rats, seems to exhibit and increasing trend with increasing ASK doses, there was no significant effect of treatment for either ASK1476 **(C)** or OXT **(D)**. Both ASK1476 **(E)** and OXT **(F)** were effective in reducing body weight gain across the dose spectrum. *Post hoc* analyses with *p*-values adjusted for multiple comparisons: *p < 0.05; **p < 0.01; ***p < 0.001; ****p < 0.0001.

**FIGURE 5:**
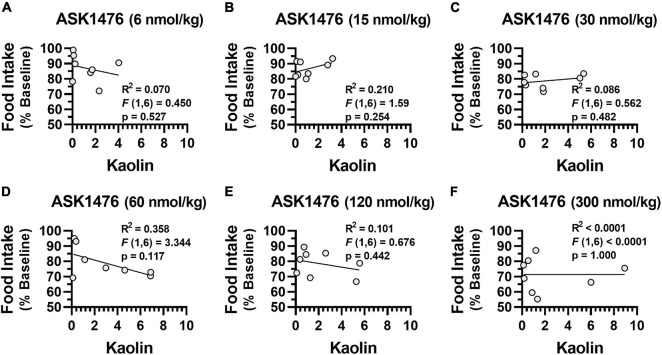
Relationship between kaolin consumption and food intake There was no significant association between kaolin consumption and baseline normalized food intake due to ASK1476 dosing at 6 nmol/kg **(A)**, 15 nmol/kg **(B)**, 30 nmol/kg **(C)**, 60 nmol/kg **(D)**, 120 nmol/kg **(E)**, and 300 nmol/kg **(F)**.

Testing the 3-day averaged dose response (i.e., 3-day saline treated baseline vs. 3-day drug treatment) of both OXT and analog ASK1476 in DIO rats, both drugs exhibited a significant interaction between the effects of treatment and dose on 3-day averaged food intake [ASK1476: *F*(2.84, 19.86) = 6.52, *p* = 0.003; OXT *F*(1.89, 13.25) = 9.49, *p* = 0.003]. Significant reductions in 3-day averaged food intake were observed starting at 15 nmol/kg (−15.20 kcal/d, 95% CI −23.46 to −6.94, *p* = 0.002) for ASK1476 treatment (Figure 4A) and at 20 nmol/kg (−11.17 kcal/d, 95% CI −19.40 to −2.94, *p* = 0.0106) for OXT (Figure 4B). Water intake was not significantly affected by either ASK1476 [*F*(1.80, 12.61) = 3.15, *p* = 0.082] or OXT treatment [*F*(1.68, 11.72) = 4.05, *p* = 0.086]. As a marker for potential nausea or visceral malaise induction during the dose escalation, rats were given access to kaolin on drug treatment days. There was no significant effect of either ASK1476 [*F*(1.80, 12.59) = 2.26, *p* = 0.148] or OXT [*F*(1.51, 10.52) = 1.73, *p* = 0.223] on kaolin intake (Figures 4C,D). However, at low doses, kaolin intake was below 4 g/d for both drugs. But only at higher doses of ASK1476 (≥ 30 nmol/kg/d) and not of OXT, there were several animals consuming kaolin > 4 g/day. Additionally, there was no correlation between food intake, as a percent of baseline, and kaolin intake (Figure 5). Similar to the observed effects on food intake, a significant interaction between treatment and dose on body weight change for both ASK1476 [*F*(2.08, 15.54) = 9.50, *p* = 0.002] and OXT [*F*(2.40, 16.79) = 16.98, *p* < 0.0001]. Significant reductions in body weight gain were observed for both ASK1476 and OXT across their respective dosing spectrums (Figures 4E,F).

Toxic effects were observed with ASK1476 following higher doses. Specifically, one rat was found dead following its third 300 nmol/kg daily dose of ASK1476 via subcutaneous injection; the cause of death was not established. Another unpublished dosing experiment with ASK1476 resulted in the death of a rat following a 150 nmol/kg dose via intraperitoneal injection. No adverse effects or tolerability issues were observed with OXT.

### Body Weight, Food Intake, Body Composition, Body Temperature, and Activity in Long-Term Study

**FIGURE 6:**
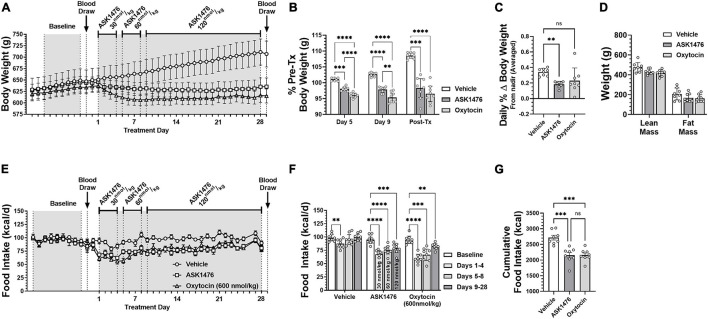
The effects of long-term (28-day) treatment with ASK1476 (starting dose 30 nmol/kg day, then increase to 60 and finally 120 nmol/kg/d) or oxytocin (OXT; 600 nmol/kg/d) on weight gain and food intake in DIO rats. Long-term reductions of body weight were observed in both ASK1476 and OXT treatment groups without compensatory weight regain **(A)**. OXT 600 nmol/kg/d yielded significantly greater reductions in body weight than ASK1476 at both the 30 nmol/kg and 60 nmol/kg doses, however, the effect of ASK1476 dosed at 120 nmol/kg/d was more comparable **(B)**. Both ASK1476 and OXT treated animals resisted compensatory weight gain throughout the 28-d treatment period with significantly lower rates of weight regain following body weight nadir **(C)**. Post-treatment body composition analyses indicated no difference in either percent lean or fat mass relative to vehicle for either OXT or ASK1476 **(D)**. ASK1476 120 nmol/kg/d was similarly effective as oxytocin 600 nmol/kg/d at attenuating caloric intake across the 28-day experiment **(E)**. Food intake remained significantly reduced across the 28-day treatment for both OXT and ASK1476 **(F)** with the two treatments yielding comparable effects on cumulative food intake **(G)**. *Post hoc* analyses with *p*-values adjusted for multiple comparisons: **p < 0.01; ***p < 0.001; ****p < 0.0001.

In the long-term experiment, robust reductions of body weight and food intake were observed in both treatment groups without compensatory weight regain. As mentioned above, starting doses were selected to yield ∼20% reduction in food intake based on the dose escalation results. ASK1476 doses were titrated during the first 8 days of treatment to a final dose of 120 nmol/kg to achieve comparable attenuation food intake with 600 nmol/kg OXT. From treatment day 9 through the end of the experiment at day 28, treatment doses were maintained to examine their durability of effects. Overall, both the 28-day ASK1476, and OXT treatments yielded stable and comparable effects on body weight relative to pre-treatment (est. difference of effect: −1.8%, 95% CI −0.9 to 4.4, *p* = 0.187) (Figures 6A,B). The average day which the body weight nadir was achieved was at treatment day 16 (median: day 17.5) for ASK1476 and day 14 for OXT with an average daily individual percent change of body weight from nadir to end of treatment of 0.18% (SD 0.04%; mean difference vs. vehicle −0.16%, 95% CI −0.28 to −0.04, *p* = 0.009) and 0.23% (SD 0.16%; mean difference vs. vehicle −0.11%, 95% CI −0.23 to 0.01, *p* = 0.069), respectively; comparatively, vehicle injection had an average daily change of body weight of 0.34% (SD 0.05%) (Figure 6C). Regarding food intake, there was a significant interaction [*F*(6, 63) = 15.38, *p* < 0.0001] between the effects of both drug treatment (vehicle, OXT, and ASK1476) and phase (baseline, drug treatment days 1–4, 5–8, and 9–28). There was an overall effect of drug treatment on 28-day cumulative food intake [*F*(2, 21) = 17.62, *p* < 0.0001], with comparable reductions due to ASK1476 (−562.7 kcal, 95% CI −837.3 to −288.2, *p* = 0.0001) and OXT (−557.1 kcal, 95% CI −831.7 to −282.6, *p* = 0.0001) treatment relative to vehicle (2,716 kcal, 95% CI 2,538–2,894) (Figures 6E,G). During the final 20 days, both ASK1476 and OXT treatments yielded significant reductions in food intake relative to baseline (ASK1476: −16.0 kcal/d, 95% CI −22.7 to −9.2, *p* = 0.001; OXT: −12.2 kcal/d, 95% CI −19.6 to −4.8, *p* = 0.004) (Figure 6F) while both the ASK1476 and OXT treated rats remained weight stable. No treatment-induced changes in gross motor activity [effect of drug: *F*(2, 10) = 1.53, *p* = 0.263; effect of phase: *F*(2.0, 20.4) = 0.40, *p* = 0.395] or body temperature [Dark cycle: effect of drug—*F*(1.1, 4.4) = 0.36, *p* = 0.596; effect of phase—*F*(1.0, 4.1) = 0.29, *p* = 0.626; Light cycle: effect of drug—*F*(1.1, 4.4) = 0.52, *p* = 0.524; effect of phase—*F*(1.0, 4.0) = 1.4, *p* = 0.302] were detected. Body composition analyses on carcasses showed indicated a significant main effect of treatment [*F*(2, 21) = 6.0, *p* = 0.009], however, there was no difference in body fat (−38.6 g, 95% CI −90.8 to 13.6, *p* = 0.181) or lean body mass (−43.2 g, 95% CI −95.4 to 9.0, *p* = 0.122) due to ASK1476 treatment (Figure 6D). While there was no difference in fat mass (−45.0 g, 95% CI −97.2 to 7.2, *p* = 0.103), lean mass was lower in OXT treated animals relative to vehicle treated animals (−57.6 g, 95% CU −109.8 to −5.4, *p* = 0.028).

### Hormone and Metabolic Parameters in Long-Term Study

Samples obtained from tail blood prior to the start of treatment showed no difference in fasting plasma leptin between groups (Table 2). At time of euthanasia following 28-day treatment, blood samples were collected via cardiac puncture. Plasma leptin [interaction of drug and treatment phase: *F*(2, 21) = 11.2, *p* = 0.001] was significantly reduced in both the ASK1476 (−6.3 ng/mL, 95% CI −9.4 to −3.3, *p* < 0.0001) and OXT (−7.53 ng/mL, 95% CI −10.6 to −4.49, *p* < 0.0001) treated animals pre- vs. post-treatment; no change was detected for vehicle treated animals. Additionally, changes in leptin correlated significantly with changes in body weight (*r* = 0.8356, *p* < 0.0001). No difference in post-treatment fasting plasma insulin or serum glucose were detected amongst the three treatment groups. Serum triglycerides were significantly lower for both drug treatment groups relative to vehicle [main effect of treatment: *F*(2, 21) = 7.3, *p* = 0.004; ASK1476: −47.38 mg/dL, 95% CI −5.7 to −89.0, *p* = 0.024; OXT: −59.9 mg/dL, 95% CI −18.2 to −101.5, *p* = 0.004] post-treatment; no difference was observed groups for total cholesterol [main effect of treatment: *F*(2, 21) = 0.64, *p* = 0.535] or HDL [main effect of treatment: *F*(2, 21) = 0.73, *p* = 0.492]. Additionally, ASK1476 treatment resulted in lower serum ALT [main effect of treatment: *F*(2, 21) = 6.0, *p* = 0.009] relative to vehicle (−7.6 U/L, 95% CI −2.1 to −13.2, *p* = 0.006) while AST was comparable amongst all three treatment groups (*p* = 0.553).

**TABLE 2:** Diet induced obese (DIO) rat measurements and blood parameters before and after 28-day treatment.

		ASK1476	Oxytocin	Vehicle	One-way ANOVA *p*-value
Baseline	Body weight (g)	645.638.2	637.139.6	650.971.3	0.867
	Avg food intake (kcal/d)	95.08.4	94.88.3	98.46.9	0.595
	Lee index	0.3230.007	0.3210.005	0.3230.008	0.805
	Leptin (ng/mL)	33.68.9	32.012.2	32.19.4	0.883
Post-treatment (after 28-day Tx)	Body weight (g)	635.554.1	615.143.2*	706.980.1	0.041
	Fat mass (g)	168.643.1	162.238.3	207.260.1	0.153
	Lean mass (g)	430.926.6	416.531.9*	474.149.2	0.015
	Leptin (μg/L)	27.210.1	24.410.4	31.910.0	0.353
	Insulin (μg/L)	4.51.8	4.42.8	3.71.1	0.539
	Glucose (mmol/L)	10.60.8	10.11.2	10.12.0	0.231
	Na (mEq/L)	144.01.8	143.91.0	142.51.3	0.081
	K (mEq/L)	4.50.3	4.60.3	4.60.4	0.461
	ALT [μmol/(sL)]	0.440.06**	0.500.09	0.570.07	0.009
	AST [μmol/(sL)]	1.320.55	1.530.62	1.260.13	0.553
	Cholest (mmol/L)	2.10.4	2.20.2	2.00.3	0.535
	Trig (mmol/L)	0.90.3*	0.80.2**	1.50.5	0.004
	HDL (mg/dL)	0.70.1	0.70.1	0.70.1	0.492

Data presented as Mean ± SD. Post hoc analyses with p-values adjusted for multiple comparisons: vs. Vehicle *p < 0.05; **p < 0.01.

Given that ASK1476 has been shown to significantly activate the V_2_R, we included assessments of serum electrolytes. There were no differences in serum sodium [main effect of treatment: *F*(2, 21) = 2.8, *p* = 0.081] or potassium [main effect of treatment: *F*(2, 21) = 0.8, *p* = 0.461] between treatment groups post-treatment (Table 2).

## Discussion

In the present study, we demonstrate that the injectable long-acting selective OXT receptor agonist ASK1476 results in similar reductions of food intake and body weight in DIO rats to native OXT, at lower doses. In model organisms, peripheral administration of OXT reduces food intake, enhances energy expenditure, and prevents or attenuates weight gain in rodent models of diet-induced obesity in rodent models of genetic forms of obesity (Morton et al., 2012), as well as in non-human primates with diet-induced obesity. As mentioned before, there are significant data in obese animals and humans demonstrating that OXT reduces food intake and body weight but that it has significant limitations as a drug. These include poor metabolic stability that translates to sub-optimal PK and pharmacodynamic (PD) properties (Ring et al., 2010; Steckler et al., 2012) as well as limited selectivity between OXTR and VRs (Postina et al., 1996; Akerlund et al., 1999; Gimpl and Fahrenholz, 2001; Manning et al., 2012; Wiśniewski et al., 2014; Snider et al., 2019). Other reported OXTR agonists suffer from similar liabilities and do not demonstrate safe and effective long-term reduction of body weight compared with OXT (Altirriba et al., 2014). Patient compliance is a major concern with respect to the durability and effectiveness of drugs used to treat chronic illnesses (Caldeira et al., 2014; Durden et al., 2017). Currently the only published weight-loss study using OXT in humans administers intranasal OXT 4 times per day (Zhang et al., 2013), which may cause compliance issues that reduce its long-term efficacy. Considering the above, one of our key aims is to develop OXT analogs with increased metabolic stability and improved PK properties (e.g., longer plasma half-life and peripheral exposure) compared to OXT to reduce dosing frequency (e.g., once daily) and improve patient compliance, long-term efficacy, and safety. In contrast to studies in humans, which required several applications of an intranasal OXT per day, we were able to show significant reductions of food intake and body weight gain following once daily sc injections of ASK1476 in rats.

The compound ASK1476 was described earlier as a lipidated analog of OXT designed to enhance binding to plasma proteins (structure published before as PF1) (Modi et al., 2016). This property allows for reduction of the free fraction of ASK1476 available for enzymatic degradation. The pharmacokinetics study protocol for ASK1476 in mice and rats was described before (Modi et al., 2016). Specifically, following iv administration in rats, the lipidated analog PF1 exhibited reduced plasma clearance and an increased steady-state volume of distribution compared to OXT, resulting in an extended half-life. Additionally, after sc administration to mice, PF1 displayed an extended half-life and an improved total maximal plasma concentration (C_max_) at 2 h post-dose further suggesting increased plasma binding profile of the molecule. Furthermore, the authors reported that the sc administration of the molecule in the depot formulation resulted in prolonged exposure in the plasma with only minimal concentrations detectable in the central compartments. Specifically, the brain to plasma concentration ratio suggesting almost exclusive compartmentalization of the molecule to plasma.

The effects of OXT are thought to be attributed to a combination of CNS and peripheral OXTRs. However, only small amounts of peripherally administered OXT are able to enter the CSF from the periphery in both non-human primate and rodent models and penetrating the blood brain barrier may not be required to see central effects (Leng and Ludwig, 2016), specifically reductions in food intake (Ho et al., 2014; Iwasaki et al., 2015). Iwasaki et al. (2015) demonstrated that systemic administration of OXT reduces food intake, in part, through a vagally-dependent mechanism. In addition, Zhang and Cai demonstrated that systemic OXT treatment may influence release of endogenous OXT within the hypothalamus (Zhang et al., 2013). Collectively, these studies suggest that systemic administration of OXT may be an effective strategy to recapitulate the well characterized effects of CNS OXT on energy homeostasis with benefit of reduced chance of unexpected off target central side effects. Given these prior findings, and assuming the low brain to plasma concentration ratio previously described in mice (Modi et al., 2016) is conserved in rats, it is reasonable to postulate that the effects observed in the presented studies may have been achieved through peripheral OXT signaling pathways, such as OXT activated vagal afferent signaling as opposed to direct central signaling. Further studies confirming ASK1476 concentrations in central compartments and the role vagal afferent signaling plays in the effects of ASK1476 treatment in rats are needed.

During the dose escalation experiment kaolin powder intake was recorded as an indicator of potential nausea and visceral malaise. While the changes in kaolin intake across the ASK1476 dosing spectrum were not significant, there were a few animals that had notably high kaolin intake. Amongst those few animals there seems to be a distinct dose effect supporting that ASK1476 treatment may result in visceral malaise in a subset of animals, an effect that was not observed in any of the OXT treated animals. As kaolin intake results can be confounded by both animal specific preference and behavior, and notable visceral malaise at therapeutic doses would be a limiting factor in the therapeutic potential of a drug, additional studies to compare kaolin intake after ASK1476 with that after vehicle alone, along with conditioned flavor avoidance testing in rodents and emetic response testing in shrews would be warranted.

PF1 did not display V_1a_R activation, instead the molecule was reported to be V_1a_R antagonist with a binding Ki of 4 nM (Modi et al., 2016). These data associated with the exclusive peripheral (vs. CNS) distribution of the molecule suggests that ASK1476 is unlikely to lead to cardiovascular or central side effects such as reductions in forebrain CNS OXTR (Insel et al., 1992; Peters et al., 2014; Freeman et al., 2018) and off-target effects second to AVPR1a binding (Freeman et al., 2018). However, the molecule was potent activator of V_2_R to show EC_50_ of 0.36 nM in the cAMP assay.

Whereas the overall profile of ASK1476 met the primary aim of our studies including the enhanced stability of the OXTR activator *in vivo*, the molecule did display a significant activation of the V_2_R. As mentioned above, the receptor plays the key role in both urine and water homeostasis. In the clinical setting, the V_2_R agonist desmopressin exhibits related side effects including hyponatremia and renal water retention. Whereas we did not observe similar adverse effects during our studies, our next round of chemical optimization is focused on designing molecules with no significant V_1a_R and V_2_R activating potential.

Besides addressing reduction of food intake, the recent identification of recruitable brown adipose tissue (BAT) in humans (van Rooijen et al., 2013) has renewed focus on developing drugs which lead to increased energy expenditure. OXT is a downstream target of the adipose tissue hormone leptin. In diet-induced obese non-human primates OXT elicits weight loss, in part, by increasing energy expenditure (Blevins et al., 2015). In contrast, reduced OXT signaling is associated with obesity and reduced energy expenditure and thermogenesis in BAT (Takayanagi et al., 2008; Kasahara et al., 2013). However, the exact mechanism on energy expenditure is poorly understood. In this study, we did not find any evidence that OXT or ASK1476 treatment led to changes in locomotor activity or body temperature. However, we were not able to perform in depth analyses of energy expenditure, such as indirect calorimetry. Post-treatment body composition did not indicate a significant difference in either lean or fat mass in ASK1476 animals relative to the vehicle (saline) treated control group. However, these animals were still growing and ASK1476 prevented weight gain compared to the steady body weight gain of the vehicle treated group. Furthermore, the drug treatment did not lead to any changes in activity or body temperature, and the ratio of lean and fat mass to total body mass was conserved between the vehicle and drug treated groups. This indicates that the caloric deficit was sufficient to attenuate the growth of lean mass but insufficient to induce loss of fat mass. Further experiments examining the changes in body composition and energy expenditure due to treatment are needed and may be better performed in adult rather than developing animals.

In summary, obesity drug intervention using OXT-based therapeutics is promising based on results in animal studies, while human data are scarce. This could be due the limitations of OXT, including short half-life. We present data using a long-acting OXT analog with improved potency to reduce food intake and body weight in DIO animals which improves on the limitations of OXT as an anti-obesity drug intervention. The improved PK profile of ASK1476 combined with its comparable efficacy and improved potency relative to OXT may allow for more favorable dosing regimens than what is achievable with the current intranasal OXT formulations. Other notable advantages include its compartmentalization to plasma attenuating the risk of central side effects and decreased risk of V_1a_R mediated cardiovascular side effects due to its antagonistic properties at that receptor. These data provide evidence that selective activation of the OXTR pathway can result in both acute and chronic beneficial metabolic effects. However, one needs to be aware that potential activation of vasopressin receptors by non-selective OXT analogs can cause unintended effects. Therefore, further optimization including development of long-acting OXTR agonists without activity at vasopressin receptors, followed by careful testing for efficacy in safety, will be important.

## Data Availability Statement

The raw data supporting the conclusions of this article will be made available by the authors, without undue reservation.

## Ethics Statement

The animal study was reviewed and approved by the Seattle Children’s Research Institute Institutional Animal Care and Use Committee.

## Author Contributions

CR, CE, AK, JB, and DS contributed to conception and design of the study. CE organized the database and performed the statistical analyses. CR wrote the first draft of the manuscript. CE, JB, and EL wrote sections of the manuscript. AK wrote the first draft for receptor binding studies. All authors contributed to manuscript revision, read, and approved the submitted version.

## Conflict of Interest

AK, DS, and RP are current employees of OXT Therapeutics and may possess stock options. JB and EL have a financial interest in OXT Therapeutics, Inc., a company developing highly specific and stable analogs of oxytocin to treat obesity and metabolic disease. The authors’ interests were reviewed and are managed by their local institutions in accordance with their conflict-of-interest policies. The remaining authors declare that the research was conducted in the absence of any commercial or financial relationships that could be construed as a potential conflict of interest.

## Publisher’s Note

All claims expressed in this article are solely those of the authors and do not necessarily represent those of their affiliated organizations, or those of the publisher, the editors and the reviewers. Any product that may be evaluated in this article, or claim that may be made by its manufacturer, is not guaranteed or endorsed by the publisher.
